# Cross-sectional survey of flavored cigarette use among adult smokers in Singapore

**DOI:** 10.18332/tid/135321

**Published:** 2021-06-03

**Authors:** Yvette van der Eijk, Xian Yi Ng, Jeong Kyu Lee

**Affiliations:** 1 Saw Swee Hock School of Public Health, National University of Singapore, Singapore

**Keywords:** survey, menthol, Asia, Singapore, flavors

## Abstract

**INTRODUCTION:**

Singapore, a city-state with a multi-ethnic Asian population, has one of the world’s largest market shares for cigarettes with added flavors, such as menthol and fruit, which increase the appeal of smoking. Little is known on the sociodemographic or smoking-related traits associated with flavored cigarette use in the Asian context.

**METHODS:**

A cross-sectional survey was conducted in January–March 2020 of 1123 Singaporean adult (aged 21–69 years) current smokers using a self-completed online or postal questionnaire. We used descriptive statistics and bivariate analyses to compare the answers of flavored and non-flavored cigarette users and fitted a multivariate logistic regression model to identify correlates of flavored cigarette use.

**RESULTS:**

Of the respondents, 85.2% reported ever use and 52.7% of respondents with a regular brand reported current use of flavored cigarettes. Older age 40–49 years (AOR= 0.63) age ≥50 years (AOR=0.60), Indian ethnicity (AOR=0.39), and a moderate (AOR=0.57) or high (AOR=0.34) dependence level were correlated with non-flavored cigarette use, while female gender (AOR=2.53) and a later initiation age (16–20 years: AOR=1.72; age ≥21 years: AOR=2.19) were correlated with flavored cigarette use.

**CONCLUSIONS:**

Consistent with findings from other countries, flavored cigarette use in Singapore is associated with being younger in age, female, of a certain ethnicity (Malay or Chinese), and having a lower nicotine dependence level.

## INTRODUCTION

Flavored cigarettes contain additional characterizing flavors such as menthol, mint, fruit or sweets. These flavors may be added to the tobacco leaf or incorporated into cigarettes in diverse forms such as scent infusions, surface coatings, threads, granules, microcapsules, or crushable capsules embedded in the filter. The last form, sold in what are commonly termed ‘capsule cigarettes’, provide an on-demand flavor which enables the user to selectively add one or more flavors to the cigarette. While flavored cigarettes have been on global markets, including Singapore’s, since at least the 1970s^1^, capsule cigarettes are a more recent development^2^, launched in Singapore in 2010^1^.

Tobacco flavors including menthol have become increasingly regulated with bans in Brazil, Ethiopia, Uganda, Senegal, Mauritania, Turkey, the European Union, Canada, and parts of the United States^3,4^. Tobacco flavor bans came in response to evidence which consistently shows that tobacco flavors, especially menthol, encourage smoking initiation^5-10^, increase the addictiveness of cigarettes^11,12^, and mislead people into thinking cigarettes are less harmful^13-16^. More recently, evidence from numerous countries has also illustrated the popularity of capsule cigarettes among young people owing to their novelty appeal, youthful designs, and wide variety of flavors^17-23^.

Singapore is a developed city-state in South-East Asia with a multi-ethnic (predominantly Chinese, Malay and Indian) Asian population. Although Singapore has a comprehensive and well-established tobacco control policy dating back to 1970^24^, Singapore has no regulations on tobacco flavors. As of 2018, Singapore’s flavored cigarette market share was one of the largest in the world, with cigarette market shares of 48% for menthol (non-capsule) cigarettes and 3% for capsule cigarettes^25^. Singapore’s menthol market grew rapidly in the 1980s and 1990s as a result of tobacco industry efforts to target young people with menthol cigarettes amidst Singapore’s strict tobacco advertising and promotions ban^1^. Since the mid 2000s, Singapore’s menthol market share has remained stable at over 40% while Singapore’s capsule cigarette market, although still small, started growing rapidly in the mid 2010s and is expected to grow further into the 2020s^25^.

Surveys of flavored cigarette use in the European Union^26^, Poland^27^, United States^28,29^, and Chile^30^, have found that smokers who use flavored cigarettes are more likely to be younger in age^26-30^, female^26-28,30^, of an ethnic minority^28^, smoking fewer cigarettes per day^26^, and more likely to perceive some cigarettes as less harmful than others^27^. However, little is known on the sociodemographic or smoking-related characteristics associated with flavored cigarette use in Singapore or the Asian context. The goal of this study was to identify sociodemographic and smokingrelated traits associated with flavored cigarette use in Singapore.

## METHODS

### Study design and population

We used cross-sectional data from 1123 adult (aged 21–69 years) current smokers who were a Singapore Citizen or Permanent Resident (PR) at the time of survey. Following the Centers for Disease Control and Prevention definition^31^, we defined ‘current smoker’ as a person who has smoked at least 100 cigarettes in their lifetime and was smoking cigarettes on a daily or non-daily basis at the time of the survey. Data were provided in a self-completed online or postal survey, which included questions on sociodemographic characteristics, current smoking, smoking at the time of initiation, use of flavored cigarettes, and perceptions of different flavor variants.

We recruited survey participants in January–March 2020 from the 2019 Singapore Smokers’ Survey (SSS) project. All SSS participants were adults, Singapore Citizens or PR, and current smokers who had agreed to be re-contacted for follow-up research. They had been recruited into the SSS from existing cohort studies: 1) the Singapore Population Health Studies (SPHS) cohort first follow-up study and Multi-Ethnic Cohort Phase 3 study; 2) the SPHS Online Panel; and 3) the National Population Health Survey 2016/2017, with further recruitment in designated smoking areas in public places, through personal contacts, and distribution of recruitment flyers.

We invited eligible participants (adult, Singapore Citizen/PR, current smoker) to participate in the survey by phone call after which they were either emailed a link to complete the questionnaire online or sent a hard copy of the questionnaire to their postal address. To increase the survey response rate, a member of the research team made a follow-up call to those who did not respond to the invitation within two weeks.

Questionnaires, as well as the participant information sheet and informed consent form shown prior to the survey, were available in English and Mandarin Chinese, the two most widely spoken languages in Singapore. The online surveys were hosted on Verint’s MySurvey, from which data were electronically imported into Microsoft Excel. Data from the postal surveys were entered manually into Excel. Each participant was reimbursed with a $5 supermarket voucher upon completion of the survey.

### Measurements

#### Flavored cigarette use at time of survey

This was assessed with three questions. First, participants were asked: ‘is there a certain brand or variant of cigarette you prefer over others?’ with response ‘yes/no’. Those who selected ‘yes’ were then asked the open-ended question ‘what is the full name of your preferred brand and variant of cigarette?’ and a multiple choice question ‘how would you describe the flavor of that brand?’ with the choice to select any of the options: 1) regular or full (tobacco) flavor; 2) light or mild; 3) menthol or mint flavored; 4) clove flavored (kretek); 5) fruit or candy flavored; and 6) flavor capsule. We grouped participants into one of two flavor categories (flavored, non-flavored) and one of five flavor subcategories (menthol, kretek, capsule, regular, light) accordingly (Table 1). Coding accuracy was checked against the open-ended answers and amended where necessary. A total of 861 participants (76.7% of all participants) were categorized as flavored (n=454) and non-flavored (n=407) current users. The remainder either indicated they did not have a preferred brand or gave inconsistent answers which could not be coded.

**Table 1:** Categories and subcategories for flavor preference

*Flavor category*	*Flavor subcategory*	*Description of subcategory*	*Code description*
Flavored	Menthol	Contains menthol/mint, may also contain other flavors such as fruit or candy. Does not contain a crushable flavor capsule.	If (3) or (5), and not (6)
	Kretek	Clove-flavored Indonesian kretek, may or may not contain menthol.	If (4)
	Capsule	Contains a crushable flavor capsule.	If (6)
Non-flavored	Regular	Does not contain added flavors and is not positioned as ‘mild’ or ‘light’.	If (1) and not (2)–(6)
	Light	Does not contain added flavors, is positioned as ‘mild’ or ‘light’.	If (2) and not (3)–(6)

Codes: 1) regular or full (tobacco) flavor; 2) light or mild; 3) menthol or mint flavored; 4) clove flavored (kretek); 5) fruit or candy flavored; and 6) flavor capsule.

#### Flavored cigarette use at time of initiation

We assessed this with three retrospective questions. First, participants were asked: ‘when you started smoking cigarettes, was there a certain brand or variant of cigarette you preferred over others?’ with response ‘yes/no’. Those who selected ‘yes’ were then asked the open-ended question ‘what was the full name of that brand and variant of cigarette?’ and a multiple choice question ‘how would you describe the flavor of that brand?’ with the choice to select any of the options: 1) regular or full (tobacco) flavor; 2) light or mild; 3) menthol or mint flavored; 4) clove flavored (kretek); 5) fruit or candy flavored; and 6) flavor capsule. We coded flavor categories and subcategories according to Table 1. A total of 526 participants (46.8% of all participants) were categorized as flavored (n=312) and non-flavored (n=214) users at time of initiation. The remainder either indicated they did not have a preferred brand at the time of initiation or gave inconsistent answers which could not be coded.

#### Sociodemographic characteristics

We collected data on the participants’ age, gender, ethnicity (Chinese, Malay, Indian, other), education level categorized as ‘primary’ (PSLE, no formal qualifications), ‘secondary’ (O/N level, NTC 3 or equivalent) ‘pre-university’ (A level, NTC 1–2 or equivalent, polytechnic or other diploma) or ‘university and above’, and monthly household income in SGD (≤$2000, $2000–$5999, $6000–$9999, ≥$10000) (SGD: 1000 Singapore dollars about US$750).

#### Dependence level

We assessed dependence level with frequency of smoking (daily, weekly, monthly, less than monthly) and Heaviness of Smoking Index (HSI). For participants who reported daily smoking, we calculated HSI score using two questions: ‘on days that you smoke, how soon after you wake up do you have your first cigarette?’ with responses ‘after 60 minutes, within 31–60 minutes, within 6–30 minutes, within 5 minutes’ and ‘on days that you smoke, how many cigarettes do you typically smoke per day?’ with responses ‘10 or fewer, 11–20, 21–30, 31 or more’. Each answer option was progressively assigned a score from 0 to 3^32^. Participants were then grouped into one of four dependence categories: 1) non-daily; 2) daily, low dependence (HSI score 0–1); 3) daily, moderate dependence (HSI score 2–4); and 4) daily, high dependence (HSI score 5–6).

#### Smoking-related behaviors

We collected data on intentions to quit (currently trying to quit, intends to quit in future, no intentions to quit, doesn’t know), age of smoking initiation, and whether participants had ever tried using flavored cigarettes, capsule cigarettes and other tobacco or nicotine products. Participants who reported using capsule cigarettes at least some of the time were asked questions on how often they burst the capsule and when during smoking they preferred to burst the capsule.

#### Smoking-related perceptions

For participants who reported using a regular brand, we collected data on their reasons for preferring this brand and perceptions of the brand’s harmfulness compared to other brands. Participants who reported using flavored cigarettes at least some of the time were asked the question ‘if flavored cigarettes were no longer available in Singapore, how do you think you would respond?’ with the choice to select any of the options: ‘I would try to quit smoking; I would switch to another cigarette; and I would try to obtain flavored cigarettes by other means’. We asked all participants to rate four different flavor variants of Marlboro, the most popular brand in Singapore, on a Likert scale in terms of overall appeal, packaging, taste, satisfaction, and harmfulness compared to other cigarettes. The variants included: 1) ‘Red’, a non-flavored regular cigarette; 2) ‘Gold’, a nonflavored light cigarette; 3) ‘Menthol’, a menthol-flavored cigarette; and 4) ‘Splash Mega Purple’, a mentholflavored cigarette with a fruit-flavored capsule.

### Statistical analysis

Statistical analyses were conducted in two stages using R version 3.6.3. First, we used descriptive statistics and bivariate analyses to summarize our sample and compare the answers of flavored and non-flavored cigarette users. We used Pearson’s chi-squared tests with Yates continuity correction and *post hoc* residuals with Bonferroni correction for categorical variables, and Student’s t-tests for continuous variables. Second, we fitted a multivariate logistic regression model to identify correlates of flavored cigarette use. In the logistic regression model, a binary current flavored cigarette use variable (flavored vs non-flavored) was regressed on potential predictors including sociodemographics (age group, gender, ethnicity, education level, household income) and smokingrelated behaviors and perceptions (dependence level, intentions to quit, age of initiation, harm perception, reasons for brand preference). To determine the predictors to be included in the regression model, we first assessed each variable in a bivariate model by setting a liberal p value (p=0.20) and retained those variables that were significant at p<0.20 for the multivariate logistic regression analysis. The regression analysis yielded adjusted odds ratios (AOR) and 95% confidence intervals (CI) to assess the relationships between the predictor variables and current flavored use.

## RESULTS

### Sociodemographics and smoking-related behaviors

Compared to the 2010 Singapore National Health Survey^33^, a nationally representative survey of smokers in Singapore, our sample was similar in terms of age distribution but had a larger proportion of females (25.6%) and Indians (13.1%). Most of our study participants were educated to pre-university (41.3%) or secondary (29.5%) level, and most (49.2%) had an intermediate monthly household income of $6000–$9999 (Table 2).

**Table 2:** Sociodemographic characteristics of participants, shown for all participants as well as those categorized as non-flavored cigarette users or flavored cigarette users

*Characteristics*	*All n (%)*	*Non-flavored n (%)*	*Flavored n (%)*
**Age (years)**			
20–29	284 (25.3)	94 (23.1)	137 (30.2)
30–39	321 (28.6)	116 (28.5)	149 (32.8)
40– 49	264 (23.5)	98 (24.1)	94 (20.7)
≥50	254 (22.6)	99 (24.3)	74 (16.3)
**Gender**			
Male	833 (74.4)	336 (83.0)	288 (63.6)
Female	286 (25.6)	69 (17.0)	164 (36.4)
**Ethnicity**			
Chinese	674 (60.0)	209 (51.4)	281 (61.9)
Malay	228 (20.3)	85 (20.9)	108 (23.8)
Indian	147 (13.1)	83 (20.4)	36 (7.9)
Other	74 (6.6)	30 (7.4)	29 (6.4)
**Education level**			
Primary^a^	66 (5.9)	20 (4.9)	16 (3.5)
Secondary^b^	331 (29.5)	127 (31.2)	118 (26.0)
Pre-university^c^	464 (41.3)	175 (43.0)	197 (43.4)
University and above	262 (23.3)	85 (20.9)	123 (27.1)
**Average monthly household income (SGD)**			
≤$2000	100 (8.9)	59 (15.9)	58 (13.9)
$2000–$5999	172 (15.3)	216 (58.1)	212 (51.0)
$6000–$9999	552 (49.2)	57 (15.3)	85 (20.4)
≥$10000	182 (16.2)	40 (10.8)	61 (14.7)

SGD: 1000 Singapore dollars about US$750.

a No formal qualifications/primary, PSLE.

b Secondary, O/N level or NTC 3 certificate or equivalent.

c A level or NTC 1–2 certificate or equivalent, polytechnic diploma, other diploma and professional qualification.

Most participants were daily smokers with a moderate dependence level (48.0%) or low dependence level (28.0%). Of all participants, 20.6% were non-daily smokers, 19.6% reported that they were attempting to quit and 19.8% were planning to quit in future, while 34.1% were unsure of their quit intentions, and 26.5% had no intention to quit.

### Use of flavored cigarettes and other tobacco products

The vast majority (85.2%) of participants reported they had ever used a flavored cigarette, while over half (55.7%) reported they had ever used a capsule cigarette. Among participants who reported using capsule cigarettes at least some of the time, most (64.3%) reported they ‘always’ burst the capsule, and most (72.8%) preferred to burst the capsule prior to smoking the cigarette.

The majority of participants (78.2%) indicated they had a regular brand or type of cigarette. For most of these participants (52.7%), this regular brand was a flavored cigarette (34.6% menthol non-capsule, 15.0% capsule, 2.0% kretek), for 25.8% it was a non-flavored regular cigarette, and for 21.5% it was a non-flavored light cigarette.

Besides cigarettes, other nicotine products participants most commonly reported having ever used were shisha (29.7%), e-cigarettes (29.0%), cigars (28.8%), and roll your own tobacco (28.3%). Flavored cigarette users were more likely than non-flavored users to have ever used e-cigarettes (37.2% vs 26.3%, p<0.001) or shisha (36.1% vs 28.5%, p=0.021), while non-flavored users were more likely to have ever used cigars (34.2% vs 26.7%, p=0.002), roll your own tobacco (32.9% vs 24.9%, p=0.012), or pipe tobacco (8.4% vs 4.6%, p=0.036).

### Predictors of current flavored cigarette use

Our regression analysis identified seven characteristics significantly associated with being a current flavored cigarette user: age, gender, ethnicity, dependence level, age of initiation, harm perception, and reasons for brand preference (Table 3).

**Table 3:** Results of multivariate logistic regression assessing correlates of flavored cigarette use

*Variable*	*AOR (95% CI)*	*p*
**Age**		
20–29	Ref.	-
30–39	0.98 (0.65–1.46)	0.90
40–49	0.63 (0.41–0.98)	0.04*
≥50	0.60 (0.37–0.97)	0.04*
**Gender**		
Male	Ref.	-
Female	2.53 (1.77–3.63)	<0.01*
**Ethnicity**		
Chinese	Ref.	-
Malay	0.94 (0.63–1.39)	0.74
Indian	0.39 (0.24–0.52)	<0.01*
Others	0.64 (0.34–1.19)	0.16
**Education level**		
Primary	1.10 (0.47–2.52)	0.83
Secondary	1.06 (0.67–1.68)	0.79
Pre-university	1.02 (0.69–1.51)	0.92
University and above	Ref.	-
**Dependence level**		
Non–daily	Ref.	-
Daily, low dependence	0.67 (0.42–1.07)	0.09
Daily, moderate dependence	0.57 (0.36–0.88)	0.01*
Daily, high dependence	0.34 (0.13–0.86)	0.02*
**Age of initiation (years)**		
≤15	Ref.	-
16–20	1.72 (1.23–2.41)	<0.01*
≥ 21	2.19 (1.33–3.66)	<0.01*
**Harm Perception**		
Less harmful	Ref.	-
Equally/more harmful	1.88 (1.12–3.16)	0.02*
**Reason for brand preference**		
It tastes better	1.30 (0.94–1.79)	0.11
It smells better	1.27(0.84–1.93)	0.26
It provides a stronger kick	0.29 (0.19–0.45)	<0.01*
It is smoother on my airways	1.37 (1.00–1.88)	0.05
It is cheaper	1.91 (1.34–2.74)	<0.01*
I started smoking that brand	0.82 (0.55–1.22)	0.33

* Statistically significant results (p<0.05). AOR: adjusted odds ratio. CI: confidence interval.

Smokers who were older, age 40–49 years (AOR=0.63; 95% CI: 0.41–0.98), ≥50 years (AOR=0.60; 95% CI: 0.37–0.97) and of Indian ethnicity (AOR=0.39; 95% CI: 0.24–0.52) were less likely to use flavored cigarettes, while females were more likely than males to use flavored cigarettes (AOR=2.53; 95% CI: 1.77–3.63). Daily smokers who reported a moderate (AOR=0.57; 95% CI: 0.36– 0.88) or high dependence level (AOR=0.34; 95% CI: 0.13–0.86) were less likely to use flavored cigarettes compared to non-daily smokers. Those who started smoking at age 16 years or later were more likely to use flavored cigarettes compared to those who started at age ≤15 years: 16–20 years (AOR=1.72; 95% CI: 1.23–2.41); ≥21 years (AOR=2.19; 95% CI: 1.33–3.66).

Finally, flavored cigarette users were more likely to perceive their brand as equally or more harmful than other brands (AOR=1.88; 95% CI: 1.12–3.16), and to prefer their brand because ‘it is cheaper’ (AOR=1.91; 95% CI: 1.34–2.74). Preferring a brand because ‘it provides a stronger kick’ was associated with being a non-flavored cigarette user (AOR=0.29; 95% CI: 0.19–0.45).

### Smoking-related perceptions

Among participants who reported having a regular brand, most (88.4%) perceived their brand as equally harmful to other brands. A further 10.2% perceived their brand as less harmful; these participants were more likely to be smokers of non-flavored light cigarettes compared to flavored or regular nonflavored cigarettes (16.8% vs 7.7% and 7.2%; p<0.006, chi-squared with adjusted residuals).

Table 4 displays the results of chi-squared tests to assess associations between perceptions of different Marlboro variants and flavored cigarette use among adult smokers. Brand recognition was high, with virtually all (99.7%) participants reporting that they had heard of the Marlboro brand. Compared to nonflavored cigarette users, flavored users were more likely to perceive the non-flavored variants (regular and light) less favorably and the flavored variants (non-capsule and capsule) more favorably in terms of look/packaging, taste, satisfaction, and overall appeal. Harm perceptions of the different variants were similar between flavored and non-flavored cigarette users.

**Table 4:** Perceptions of different flavor variants as reported by non-flavored (NF) and flavored (F) cigarette users

*Response*	*Red (non-flavored, regular)*		*Gold (non-flavored, light)*		*Menthol (non-capsule)*		*Splash mega purple (capsule)*	
	*NF, n (%)*	*F, n (%)*	*NF, n (%)*	*F, n (%)*	*NF, n (%)*	*F, n (%)*	*NF, n (%)*	*F, n (%)*
**‘I like the look of the packaging and cigarette’**								
Strongly agree	52 (12.8)**	33 (7.3)	82 (20.1)	59 (13.1)	51 (12.5)	77 (17.0)	78 (19.2)	111 (24.6)
Somewhat agree	213 (52.3)	218 (48.0)	207 (50.9)	235 (52.0)	176 (43.2)	245 (54.1)**	152 (37.3)	219 (48.5)**
Somewhat disagree	92 (22.6)	140 (30.8)***	84 (20.6)	110 (24.3)	123 (30.2)***	94 (20.8)	99 (24.3)***	77 (17.0)
Strongly disagree	50 (12.3)	63 (13.9)	34 (8.4)	48 (10.6)	57 (14.0)**	37 (8.2)	78 (19.2)***	45 (10.0)
**‘This looks like the kind of cigarette I would smoke’**								
Strongly agree	62 (15.2)***	16 (3.5)	95 (23.3)***	20 (4.4)	24 (5.9)	96 (21.2)***	26 (6.4)	93 (20.6)***
Somewhat agree	195 (47.9)***	108 (23.8)	160 (39.3)	161 (35.6)	112 (27.5)	256 (56.5)***	96 (23.6)	205 (45.4)***
Somewhat disagree	85 (20.9)	164 (36.1)***	108 (26.5)	168 (37.2)***	170 (41.8)***	67 (14.8)	152 (37.3)***	95 (21.0)
Strongly disagree	65 (16.0)	166 (36.6)***	44 (10.8)	103 (22.8)***	101 (24.8)***	34 (7.5)	133 (32.7)***	59 (13.1)
**‘This cigarette looks like it would have a nice taste’**								
Strongly agree	51 (12.5)***	17 (3.7)	60 (14.7)***	19 (4.2)	24 (5.9)	78 (17.2)***	34 (8.4)	91 (20.1)***
Somewhat agree	181 (44.5)***	105 (23.1)	176 (43.2)	159 (35.2)	139 (34.2)	239 (52.8)***	134 (32.9)	219 (48.5)***
Somewhat disagree	112 (27.5)	189 (41.6)***	118 (29.0)	174 (38.5)***	153 (37.6)***	99 (21.9)	130 (31.9)***	90 (19.9)
Strongly disagree	63 (15.5)	143 (31.5)***	53 (13.0)	100 (22.1)***	91 (22.4)***	37 (8.2)	109 (26.8)***	52 (11.5)
**‘This cigarette looks like it would be satisfying’**								
Strongly agree	54 (13.3)***	20 (4.4)	62 (15.2)***	16 (3.5)	21 (5.2)	70 (15.5)***	26 (6.4)	70 (15.5)***
Somewhat agree	195 (47.9)***	137 (30.2)	165 (40.5)	161 (35.6)	131 (32.2)	239 (52.8)***	119 (29.2)	221 (48.9)***
Somewhat disagree	99 (24.3)	170 (37.4)***	120 (29.5)	176 (38.9)***	158 (38.8)***	105 (23.2)	144 (35.4)***	101 (22.3)
Strongly disagree	59 (14.5)	127 (28.0)***	60 (14.7)	99 (21.9)***	97 (23.8)***	39 (8.6)	118 (29.0)***	60 (13.3)
**‘This cigarette looks less harmful than other cigarettes’**								
Strongly agree	8 (2.0)	14 (3.1)	18 (4.4)	14 (3.1)	8 (2.0)	20 (4.4)	10 (2.5)	20 (4.4)
Somewhat agree	50 (12.3)***	28 (6.2)	90 (22.1)	85 (18.8)	53 (13.0)	78 (17.2)	55 (13.5)	69 (15.3)
Somewhat disagree	134 (32.9)	149 (32.8)	118 (29.0)	149 (33.0)	153 (37.6)	157 (34.7)	138 (33.9)	164 (36.3)
Strongly disagree	215 (52.8)	263 (57.9)	181 (44.5)	204 (45.1)	193 (47.4)	198 (43.7)	204 (50.1)	199 (44.0)

Significant differences are indicated as: *p<0.05, **p<0.01 or ***p<0.001; chi-squared test with Bonferroni correction and adjusted residuals *post hoc*.

Participants who reported using flavored cigarettes ‘some of the time’, ‘most of the time’ or ‘always’ were asked how they would respond if flavored cigarettes were no longer available in Singapore. In all, 67.7% reported they would switch to another cigarette, 32.0% would try to quit, and 18.3% would try to obtain them by other means.

### Smoking initiation

The average age of smoking initiation among participants was 17 years (median 17 years, mean 16.9 years). The vast majority (86.4%) had started smoking by the age of 20 years and virtually all (97.5%) by 25 years. Less than half (48.3%) of participants retrospectively recalled having a regular brand at initiation. This low proportion may be due to low levels of recall or low levels of brand loyalty at the smoking initiation stage.

Among participants who reported having a regular brand at initiation, the majority (57.5%) initiated with a flavored cigarette. Female participants were, compared with males, more likely to have started smoking with a flavored cigarette (73.4% vs 47.0%; p<0.001, chi-squared). Those who started with a flavored cigarette were, compared to those who started with a non-flavored cigarette, more likely to report they preferred their starting brand because it was ‘smoother on my airways’ (35.6% vs 16.8%; p<0.001, chi-squared), ‘more interesting’ (8.0% vs 2.3%; p=0.01, chi-squared), ‘smelled better’ (18.3% vs 10.3%; p=0.017, chi-squared) and ‘tasted better’ (55.1% vs 45.3%; p=0.034, chi-squared). Those who started with a non-flavored cigarette were more likely to report they preferred their starting brand because ‘it provided a stronger kick’ (25.2% vs 11.5%; p<0.001, chi-squared).

Across different age groups, younger participants were more likely than older participants to have initiated smoking with a flavored cigarette: 63.9% of those aged 20–29 years, 58.1% of those aged 30–39 years, and 52.3% of those aged 40–49 years initiated with flavored cigarettes, compared to 39.7% of participants aged ≥50 years (p<0.004, chisquared with adjusted residuals). After categorizing participants by the decade in which they started smoking, we found that those who started smoking in more recent decades were more likely to have initiated with a flavored cigarette; 82.6% of those who started smoking in the 2010s started with a flavored cigarette, compared to 68.6% in the 2000s, 55.8% in the 1990s, and 35.9% in 1960–1980.

Of those who initiated with a flavored cigarette, 76.0% were still using a flavored cigarette at the time of survey. Of those who initiated with a non-flavored cigarette, 74.0% were still using a non-flavored cigarette at the time of survey. Thus, for both flavored and non-flavored cigarettes, the ‘switching out’ rate from initiation to the time of survey was around 1 in 4.

## DISCUSSION

This study is, to our knowledge, the first to identify correlates of flavored cigarette use in a multi-ethnic Asian population. In our sample of Singapore smokers, the prevalence of flavored cigarette use was high with 85.2% of respondents reporting ever use and 52.7% of respondents with a regular brand reporting current use of flavored cigarettes. This estimate is higher than the prevalence of flavored cigarette use reported among smokers in studies from the United States^28^ and the European Union^26^. It is also consistent with market data which show that Singapore’s flavored cigarette market share is higher than that of most other countries^25^. Use of flavor capsule cigarettes was also prevalent in our sample, with 55.7% of respondents reporting ever use and 15.0% of respondents with a regular brand reporting current use.

We found that current use of flavored cigarettes in Singapore smokers was correlated with being female or of non-Indian (Chinese or Malay) ethnicity. Female smokers were also more likely to have initiated with flavored cigarettes. This is consistent with studies from the European Union^26,27^, the United States^28^, and Chile^30^, which found higher rates of flavored tobacco use in females, and a study from the United States which shows a higher prevalence of flavored tobacco use in certain ethnic groups (African and Hispanic)^28^. These differences may be a result of the tobacco industry’s targeted marketing of flavored cigarettes^7,8,14^, which has targeted females and specific ethnic groups in Singapore^1^, as well as variations in the taste and other sensory preferences between different genders or ethnic groups^14,34^. Our findings also suggest that, in the Asian context, regulations on tobacco flavors may be especially effective in deterring smoking among females and people of Malay or Chinese ethnicity, and that smoking prevention efforts may need to adopt a more gender-sensitive or culturally sensitive approach.

Consistent with findings from the European Union^26,27^, the United States^28^, and Chile^30^, we also found that, in Singapore, current use of flavored cigarettes is correlated with being younger in age (20–39 years), having initiated at a later age, and having a lower nicotine dependence level. In addition, smokers who were younger in age or who initiated smoking more recently were more likely to have started smoking with a flavored cigarette. Put together, initiation with flavored cigarettes seems to have become more prevalent over time among our survey participants. This could be a reflection of market trends which show that Singapore’s menthol (non-capsule) cigarette market started growing in the 1980s and 1990s^1^, stabilizing in the late 2000s to its current share of around 48%^25^. This stable trend, together with our observation of the low switching rates between flavored and non-flavored cigarettes, also suggests that the majority of smokers in our sample who initiated smoking with flavored cigarettes continued to use flavored cigarettes in later life.

Flavored cigarettes, especially those with menthol flavor, encourage smoking initiation as they mask the harshness of tobacco smoke^5-10^, are sold in novelty flavors that appeal to young people^17-23^, and may be perceived as less harmful^13-16^. Among our Singapore respondents, flavored cigarette users were more likely to prefer their brand for the cheaper price and, at initiation, for the novelty, smell, taste, and smoothness on the airways. While they did not rate flavored cigarettes as less harmful than other brands, it is worth noting that ‘smoothness’ and ‘mild taste’ may be indirectly associated with a perception of reduced harm^35^, which may have influenced respondents’ perceptions of harmfulness at the smoking initiation stage. In most flavored cigarettes, smoothness and mild taste are elicited by menthol which acts as a local anaesthetic in the throat^16^.

Finally, among respondents who regularly used flavored cigarettes, almost a third (32.0%) reported they would try to quit if flavored cigarettes were no longer available in Singapore. Based on this result, we would expect a substantial increase in quit attempts following the implementation of a tobacco flavors ban in Singapore. However, the original intentions reported prior to a flavors ban may not reflect the observed behaviors following its implementation. Prior to implementation of a tobacco flavors ban in Ontario, Canada, 15% had reported that they would quit, whereas after the ban, almost twice that proportion (29%) had attempted to quit^3^. The impact on cessation rate is also likely to vary depending on the breadth of the flavors ban and the size of the pre-existing flavors market. In the European Union, where smoking cessation rates were not significantly impacted by a flavors ban^36^, tobacco companies had launched new products such as flavored inserts and cigarillos to discourage quitting among flavored cigarette users^37,38^. Additionally, the pre-existing market share of flavored cigarettes in the European countries was, compared to Singapore’s, far smaller, ranging from 0.4%–12.4%^26^, making significant differences in quitting rates harder to detect.

### Limitations

Our study findings should be carefully interpreted with the limitations of the study. Although our study sample was similar to the 2010 National Health Survey in terms of sociodemographic characteristics^33^, our sample is not representative of the Singapore population and, as such, we were unable to obtain a more reliable estimate of flavored cigarette use in Singapore. In addition, causal relationships cannot be determined due to the nature of a cross-sectional study design. To address these limitations, future research would utilize a longitudinal cohort design with a rigorous representative sampling procedure. Our sample included current smokers aged ≥21 years; hence our results cannot provide detailed insight into the role of flavors in smoking initiation. Finally, the study relied upon self-reported data from participants who agreed to be re-contacted for follow-up research, collected online or by post; door to door surveys were not possible due to the COVID-19 social distancing measures. This may influence the results as a result of selection bias, social desirability bias, or issues with the retrospective recall of answers related to smoking initiation. Despite these limitations, the study provides important insights into the characteristics and correlates of flavored cigarette use in a multiethnic, urban Asian setting.

## CONCLUSIONS

In Singapore, flavored cigarette use is associated with being female and having a later initiation age, while non-flavored cigarette use is associated with Indian ethnicity, older age, and having a moderate or high nicotine dependence level.
